# Prognostic Significance of Signet Ring Cells in Gastric Cancer: The Higher Proportion, The Better Survival

**DOI:** 10.3389/fonc.2021.713587

**Published:** 2021-11-09

**Authors:** Yang Li, Yuxin Zhong, Quan Xu, Zhikai Zhu, Yantao Tian

**Affiliations:** ^1^ Department of Pancreatic and Gastric Surgery, National Cancer Center/National Clinical Research Center for Cancer/Cancer Hospital, Chinese Academy of Medical Sciences and Peking Union Medical College, Beijing, China; ^2^ Center for Big Data, National Clinical Research Center for Neurological Diseases, Beijing Tiantan Hospital, Capital Medical University, Beijing, China

**Keywords:** gastric cancer, signet ring cell, cancer survival, prognosis, treatment

## Abstract

**Background:**

Due to the fact that the definition of gastric signet ring cell cancer (GSRC) was still controversial in the past decades, the prognosis affected by the proportion of signet ring cells within gastric cancer is uncertain. This study compared the clinicopathological features and prognosis of GSRC with the various proportions of signet ring cells.

**Methods:**

We collected GSRC cases without metastasis who underwent curative (R0) resection between 2011 and 2018. Individuals who were in the low-proportion signet ring cell group (LSRC, <50%) were matched to those who were in the high-proportion signet ring cell group (HSRC, >50%) through propensity score matching (1:1). We used Cox proportional hazard regression to calculate the adjusted hazard ratios (HR) and 95% confidence intervals (CI) and explored interactions with gender and stage.

**Results:**

We had 1:1 matched individuals including 231 cases from the LSRC group and 231 cases from the HSRC group. Patients with HSRC had a significantly higher overall survival rate in the multivariable model (aHR = 0.56, 95%CI = 0.38, 0.84) compared with those with LSRC. The association of HSRC appeared to be more substantial among individuals at early stage and N0 stage (*p*-interaction < 0.01).

**Conclusions:**

This study confirms that GSRC with different proportions of signet ring cells could affect the survival of the patient. Further clinical studies should be developed in the future to provide an appropriate treatment strategy for GSRC.

## Introduction

Gastric cancer (GC) is one of the most common causes of cancer-related mortality all over the world, with an estimated 1,033,000 new cases and 780,000 deaths in 2018 (1–3). In the past decades, a steady decline in the incidence of non-cardia intestinal GC has been observed in many parts of the world, which was caused by the decreased prevalence of *Helicobacter pylori* (4–6), while a steady increase in the diffuse type of GC was seen during this period, which was driven by the increase in gastric signet ring cell carcinoma (GSRC) (7). As a special entity of gastric cancer, gastric signet ring cell carcinoma is an uncommon pathological type. The incidence of GSRC in Asia, Europe, and the United States accounted for 35% of all adenocarcinomas (8).

GSRC, a poorly cohesive carcinoma, is composed predominantly or exclusively of signet ring cells, which are characterized by the accumulation of abundant intracellular mucin with a compressed and eccentrically placed nucleus to the cell, presenting the appearance of a signet ring (9). Several studies have demonstrated that GSRC has a better outcome with a lower rate of lymph node metastasis at the early stage, while it has a worse prognosis in advanced stages (10, 11). However, research conclusions on the survival outcome of GSRC remain inconsistent, for example, there was no significant difference in the 5-year overall survival between GSRC and gastric mucinous adenocarcinoma at stages I, II, and III (*P* > 0.05) (12). The reason for the conflicting prognostic results of signet ring cell carcinoma appears to be the lack of commonly standardized GSRC definitions. According to WHO standard 2010, if the percentage of signet ring cells is predominant (>50%), GC can be recognized as GSRC, while the cutoff percentage is 90% according to the European Chapter of International Gastric Cancer Association 2019 (9, 13). Meanwhile, the diagnosis of signet ring cell proportion in GSRC may vary widely among clinicians in different countries (14). These classifications were not supported by clear and adequate evidence to explain the differences of GSRC in clinicopathological features and prognosis worldwide.

The standardization of subgroup classifications is a critical step to precisely assess epidemiological tendencies, to allow estimating the prognosis and response to pre/postoperative chemotherapy of patients with GSRC, and to design tailored treatment strategies. Therefore, we conducted a study on the effect of the proportion of signet ring cells on the overall survival outcome in GC to disentangle the mentioned inconsistency for GSRC prognosis.

## Materials and Methods

### Study Population

We collected a total of 1,069 GSRC cases who underwent curative (R0) resections with total or subtotal gastrectomy and retrieved their corresponding clinicopathological characteristics from January 2011 to December 2018. Patients with metastasis were excluded. Subtotal gastrectomy was performed for distal or middle third GC, while total gastrectomy was performed for proximal third GC. Based on the National Comprehensive Cancer Network Clinical Practice Guidelines, standard D2 lymph node dissection was performed in patients with curative intent (15). The follow-up data were prospectively collected and regularly updated every 3 months by surgeons after surgery. The overall survival was defined from the date of surgery to the date of death or the end of follow-up (April 30, 2020).

Ethical approval was obtained through the Independent Ethics Committee of the National Cancer Center/Cancer Hospital, Chinese Academy of Medical Science, and Peking Union Medical College, and all participants gave written informed consent.

### Proportion of Signet Ring Cells

The amount of signet ring cells in histological specimens was independently confirmed by two experienced pathologists according to the definition of signet ring cells, which is cells with ample cytoplasmic mucin which appears to be optically clear on hematoxylin and eosin staining and has an eccentrically placed nucleus. The pathologists reported the percentage of signet ring cell volume as compared with the total volume of the tumor cells. The amount of signet ring cells was coded into four categories as follows: (a) minority (<10% signet ring cells), (b) partialness (10–50% signet ring cells), (c) majority (50–90% signet ring cells), and (d) total (>90% signet ring cells), considering the classification by the European Chapter of International Gastric Cancer Association, WHO standard 2010, and previous experience. As shown in **Supplementary Figure S1**, we conducted a pairwise comparison of four categories. A significant difference was observed on survival between the partialness group (10–50%) and the majority group (50 90%) as well as between the minority group (<10%) and the majority group (50–90%). As for the other four groups (A, C, D, and F in **Supplementary Figure S1**), no differences were observed (*p* > 0.05). Therefore, we merged the two groups at both ends and divided the exposure into low-proportion signet ring cell group (LSRC, <50% signet ring cells) and high-proportion signet ring cell group (HSRC, >50% signet ring cells).

### Definitions of Variables

Demographic characteristics included age and gender. Age was treated as an ordinal variable: young (≤50 years), middle-aged (50–60 years), and elderly (≥60). Tumor site was classified as upper (cardia, fundus, gastroesophageal junction), middle (body, lesser/greater curvature), and lower (antrum, pylorus) part of the stomach and the entire stomach. Tumor size was divided by median (≤4 and >4 cm). The microscopic features of histology, pathology, and cell differentiation were analyzed according to tumor differentiation (poorly differentiated and moderately differentiated), Laurén classification (intestinal type, diffuse type, and mixed type) (16), nerve invasion, and lymphatic vessel invasion. The staging systems were based on the 8th edition of the American Joint Committee on Cancer TNM classification (17). The cutoff points of lymph nodes removed were 16 and 30, which can be enough to evaluate the nodal stage and prognosis of GSRC patients (17). Adjuvant chemotherapy (yes or no) was also included. All demographic and clinicopathological variables included in our analyses were selected based on previously published articles and *a priori* knowledge regarding the classification.

### Statistical Analysis

No statistical method was used to handle missing data. Frequency (*N*) and column proportions (%) were calculated for all demographic and clinicopathological variables. The distribution of variables which differed by the proportion of signet ring cells was compared by Pearson’s chi-square tests, and we found that the distribution of most variables significantly differed. In order to reduce potential selection biases and achieve the comparability of groups, propensity score matching (PSM) was performed to make two groups comparable in our study.

Individuals who were LSRC were matched to those who were HSRC through PSM (1:1) on the basis of baseline covariates, including age, tumor site, histology differentiation, nerve invasion, and stage (18). Two comparable risk groups were 1:1 matched by using nearest-neighbor matching with no replacement and a caliper of 0.03. If no covariates had propensity scores that lay within the indicated caliper distance, that covariate was removed from the matching sample (19). Patients were matched for any significant differences seen between the two groups with respect to demographics and clinicopathological characteristics. We used the “psestimate” command to select covariates and to include in the estimation function of the propensity score proposed by Imbens and Rubin (2015) (20). We assessed the balance in the distribution of covariates before and after matching using imbalance testing. Pairs were created, such that the matched covariates had comparable values of propensity scores (21). This strategy allowed the inclusion of the largest possible, however comparable, LSRC and HSRC groups. Further analyses were conducted after matching the groups.

When the proportional hazard assumption was conducted, we used Cox proportional hazard regression to calculate the adjusted hazard ratios (HR) and 95% confidence intervals (CI) for the effect; the model was adjusted for the following potential confounders: age (continuous), gender, tumor site, tumor size, histology differentiation, Lauren type, nerve invasion, lymphatic vessel invasion, stage at diagnosis, lymph nodes removed, and adjuvant chemotherapy. A subgroup analysis was conducted by gender and stage to explore if the impact of signet ring cell proportion is stronger in certain groups. Survival curves were estimated by the Kaplan–Meier method for matched overall population, stage subgroup (early *vs*. advanced), and lymph node metastasis (N0 *vs*. N+) subgroup. We further analyzed the risk of mortality in four groups of signet ring cell proportion (<10%, 10–50%, 50–90%, >90%) after PSM and conducted tests for trends by the treated signet ring cell proportion as a continuous variable in the model.

For the current analysis, two-sided *p*-values <0.05 were considered to be statistically significant. All statistical analyses and figures were performed with Stata 15.0 (College Station, TX: StataCorp, LLP).

## Results

Through a median follow-up period of 4.1 years (interquartile range, 2.4–5.9 years), a total of 1,069 (830 LSRC group and 239 HSRC group) GSRC patients were included. For the current study, we selected propensity score-matched (1:1) individuals, including 231 cases from LSRC group and 231 cases from HSRC group.

**Table 1** presents the overall distribution of demographic and clinicopathologic characteristics within the overall and included study population. Among the overall population, Pearson’s chi-square tests indicated that the distributions of age, tumor site, Laurén type, nerves invasion, lymphatic vessel invasion, stage at diagnosis, and adjuvant chemotherapy differed by the proportion of signet ring cells (*p* < 0.05). After matching, most covariates, including age, gender, tumor site, tumor size, histology differentiation, nerve invasion, lymphatic vessel invasion, and stage at diagnosis, were comparable between the two groups, although the diffuse type (83.1%), >30 lymph nodes removed (55.8%), and no adjuvant chemotherapy (66.2%) were more frequently observed in HSRC.

**Table 1:** Characteristics of 1,069 stages I–III gastric cancer patients.

Characteristics	Overall (*N* = 1,069)	Before matching (*N* = 1,069)			After matching (*N* = 462)		
		LSRC (*N* = 830)	HSRC (*N* = 239)	*P*-value^a^	LSRC (*N* = 231)	HSRC (*N* = 231)	*P*-value^a^
		*N* (%)	*N* (%)		*N* (%)	*N* (%)	
Age (year)				**<0.01**			0.70
≤50	332 (31.1)	241 (29.0)	91 (38.1)		83 (35.9)	91 (39.4)	
50–60	307 (28.7)	235 (28.3)	72 (30.1)		70 (30.3)	69 (29.9)	
≥60	430 (40.2)	354 (42.7)	76 (31.8)		78 (33.8)	71 (30.7)	
Gender				0.43			0.85
Male	694 (64.9)	544 (65.5)	150 (62.8)		142 (61.5)	144 (62.3)	
Female	375 (35.1)	286 (34.5)	89 (37.2)		89 (38.5)	87 (37.7)	
Tumor site^b^				**<0.01**			0.97
Upper	223 (20.9)	191 (23.0)	32 (13.4)		32 (13.9)	29 (12.5)	
Middle	271 (25.4)	203 (24.5)	68 (28.5)		64 (27.7)	68 (29.4)	
Lower	521 (48.7)	391 (47.1)	130 (54.4)		129 (55.8)	128 (55.4)	
Entire	54 (5.1)	45 (5.4)	9 (3.8)		6 (2.6)	6 (2.6)	
Tumor size				0.11			0.10
≤4	595 (55.7)	451 (54.3)	144 (60.3)		158 (68.4)	141 (61.0)	
>4	474 (44.3)	379 (45.7)	95 (39.8)		73 (31.6)	90 (39.0)	
Histology differentiation				0.62			0.28
Poorly	792 (74.1)	612 (73.7)	180 (75.3)		170 (73.6)	180 (77.9)	
Moderately	277 (25.9)	218 (26.3)	59 (24.7)		61 (26.4)	51 (22.1)	
Lauren type				**<0.01**			**<0.01**
Intestinal	47 (4.4)	46 (5.5)	1 (0.4)		9 (3.9)	1 (0.4)	
Diffused	700 (65.5)	500 (60.2)	200 (83.7)		162 (70.1)	192 (83.1)	
Mixed	268 (25.1)	250 (30.1)	18 (7.5)		50 (21.7)	18 (7.8)	
Not reported	54 (5.1)	34 (4.1)	20 (8.4)		10 (4.3)	20 (8.7)	
Nerve invasion				**<0.01**			0.77
Yes	532 (49.8)	450 (54.2)	82 (34.3)		85 (36.8)	80 (34.6)	
No	267 (25.0)	188 (22.7)	79 (33.1)		80 (34.6)	78 (33.8)	
Not reported	270 (25.3)	192 (23.1)	78 (32.6)		66 (28.6)	73 (31.6)	
Lymphatic vessel invasion				**<0.01**			0.82
Yes	396 (37.0)	328 (39.5)	68 (28.5)		70 (30.3)	65 (28.1)	
No	364 (34.1)	273 (32.9)	91 (38.1)		85 (36.8)	91 (39.4)	
Not reported	309 (28.9)	229 (27.9)	80 (33.5)		76 (32.9)	75 (32.5)	
AJCC 8th stage at diagnosis				**<0.01**			0.31
Stage Ia	272 (25.4)	174 (21.0)	98 (41.0)		83 (35.9)	97 (42.0)	
Stage Ib	97 (9.1)	78 (9.4)	19 (8.0)		27 (11.7)	18 (7.8)	
Stage IIa	108 (10.1)	94 (11.3)	14 (5.9)		14 (6.1)	13 (5.6)	
Stage IIb	130 (12.2)	107 (12.9)	23 (9.6)		18 (7.8)	23 (10.0)	
Stage IIIa	162 (15.2)	135 (16.3)	27 (11.3)		42 (18.2)	27 (11.7)	
Stage IIIb	155 (14.5)	120 (14.5)	35 (14.6)		29 (12.6)	33 (14.3)	
Stage IIIc	145 (13.6)	122 (14.7)	23 (9.6)		18 (7.8)	20 (8.7)	
Lymph nodes removed				0.13			**<0.01**
1–16	37 (3.5)	33 (4.0)	4 (1.7)		1 (0.4)	4 (1.7)	
17–30	484 (45.3)	381 (45.9)	103 (43.1)		130 (56.3)	98 (42.4)	
>30	548 (51.3)	416 (50.1)	132 (55.2)		100 (43.3)	129 (55.8)	
Adjuvant chemotherapy				**<0.01**			**<0.01**
Yes	367 (34.3)	309 (37.2)	58 (24.3)		79 (34.2)	55 (23.8)	
No	593 (55.5)	436 (52.5)	157 (65.7)		113 (48.9)	153 (66.2)	
Unknown	109 (10.2)	85 (10.2)	24 (10.0)		39 (16.9)	23 (10.0)	

Boldface indicates statistical significance (P < 0.05). Column percentage was reported, and percentage can differ slightly from 100% because of rounding.

LSRC, low proportion of signet ring cell in gastric cancer; HSRC, high proportion of signet ring cell in gastric cancer.

a χ^2^ test was used to compare the distribution of variables differed by the proportion of signet ring cells.

b Tumor site was divided by the upper (cardia, fundus, gastroesophageal junction), middle (body, lesser/greater curvature), and lower (antrum, pylorus) parts of the stomach and the entire stomach.

**Figure 1:**
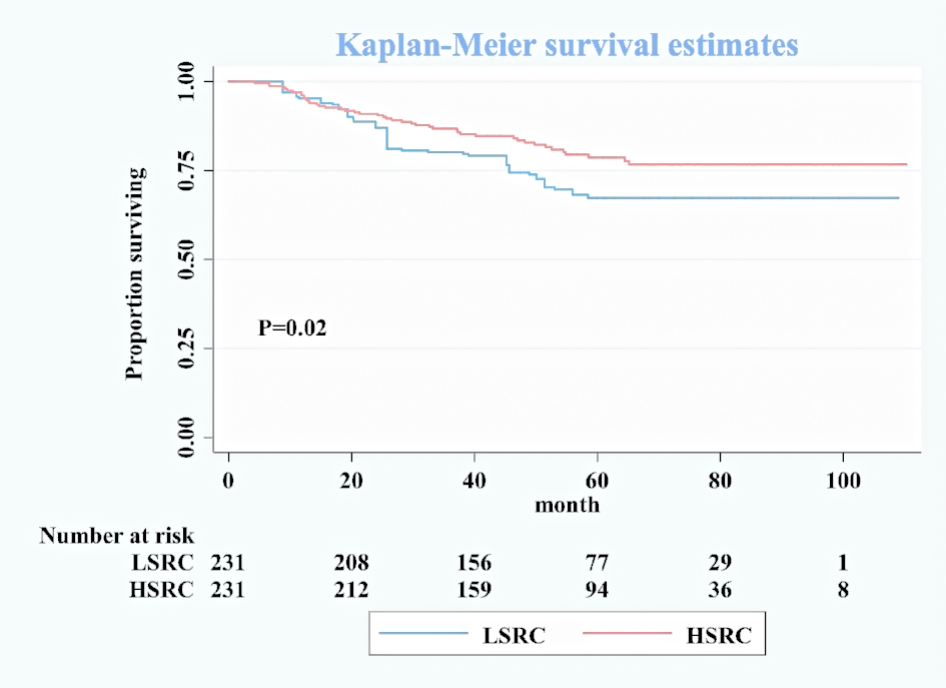
Kaplan–Meier survival curves for overall patients between low-proportion signet ring cells and high-proportion signet ring cells after matching.

The Cox proportional hazard regression model depicted in **Table 2** showed that the HSRC group, as compared with LSRC, had a better overall survival in the unadjusted model (cHR = 0.65, 95%CI = 0.45, 0.95). When adjusted for other variables, the association remained robust (aHR = 0.56, 95%CI = 0.38, 0.84). For the four groups of signet ring cell proportion, more proportions of SRC were associated with reduced mortality (HR_10–50%_ *_vs_*_. 10%_ = 0.47, 95%CI = 0.23, 0.99; HR_50–90%_ *_vs_*_. 10%_ = 0.25, 95%CI = 0.12, 0.53; SRC_>90%_ *_vs_*_. 10%_ = 0.22, 95%CI = 0.08, 0.63). The significant *p*-value trend showed the higher proportion and the better survival in **Table 2**. The survival curves in **Figure 1** show the survival probability for patients with LSRC and HSRC. The overall survival of the HSRC group was significantly longer than that of the LSRC group of patients (*P* = 0.02).

**Table 2:** Risk of mortality according to the clinical pathology among GC patients from Cox regression analysis.

Variable	After matching (*N* = 462)		
	cHR^a^ (95%CI)	aHR^a^ (95%CI)	*P*-interaction
Two proportions of signet ring cell			
LSRC	REF	REF	/
HSRC	**0.65 (0.45, 0.95)**	**0.56 (0.38, 0.84)**	
Subgroup analyses			
Gender			**<0.01**
Male	0.85 (0.54, 1.33)	**0.49 (0.28, 0.84)**	
Female	**0.35 (0.17, 0.73)**	0.50 (0.19, 1.33)	
Stage at diagnosis^b^			**<0.01**
Early	**0.21 (0.09, 0.49)**	**0.10 (0.03, 0.32)**	
Advanced	1.19 (0.76, 1.86)	0.95 (0.57, 1.58)	
Four proportions of SRC			
<10%	REF	REF	
10–50%	**0.34 (0.19, 0.58)**	**0.47 (0.23, 0.99)**	
50–90%	**0.27 (0.15, 0.49)**	**0.25 (0.12, 0.53)**	
>90%	**0.21 (0.09, 0.51)**	**0.22 (0.08, 0.63)**	

p-trend < 0.01.

Boldface indicates statistical significance (P < 0.05).

aHR, adjusted hazard ratio; cHR, crude hazard ratio; LSRC, low proportion of signet ring cell in gastric cancer; HSRC, high proportion of signet ring cell in gastric cancer.

a Cox proportional hazard regression was used to calculate the crude and adjusted hazard ratios and 95% confidence intervals.

b Early stage refers to stage I and stage II; advanced stage refers to stage III.

**Figure 2:**
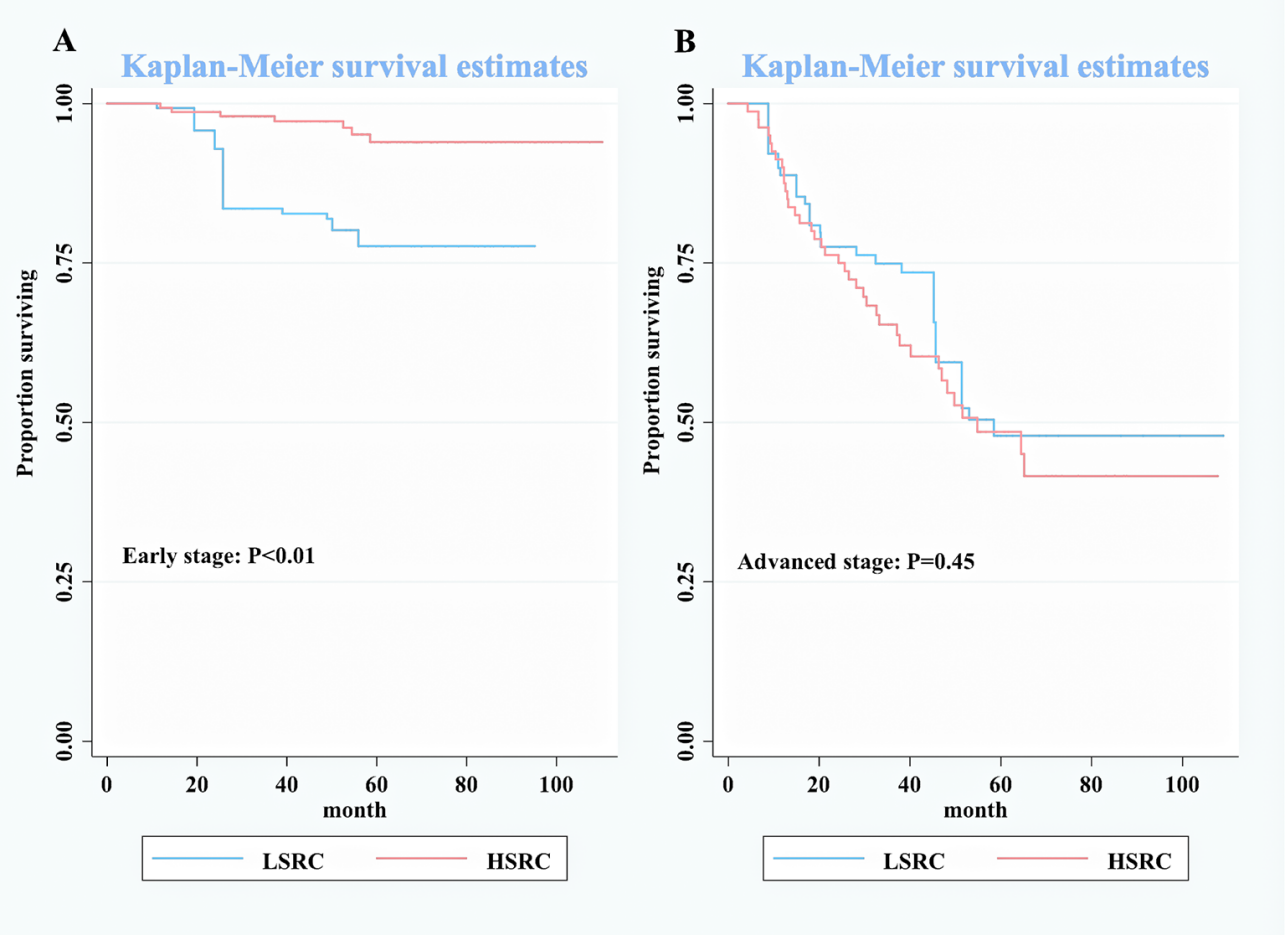
Kaplan–Meier survival curves for patients by stage between low-proportion signet ring cells and high-proportion signet ring cells. **(A)** Early stage. **(B)** Advanced stage.

**Figure 3:**
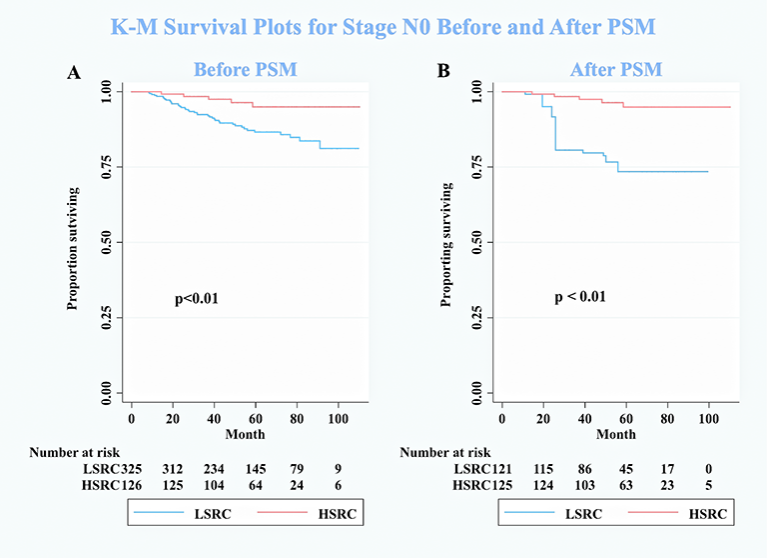
Kaplan–Meier survival curves for patients on stage N0 between low-proportion signet ring cells and high-proportion signet ring cells. **(A)** Before PSM. **(B)** After PSM. N0 means no positive lymph nodes. Conversely, N+ means extensive lymphatic involvement, which is a higher pathologic N stage.

**Figure 4:**
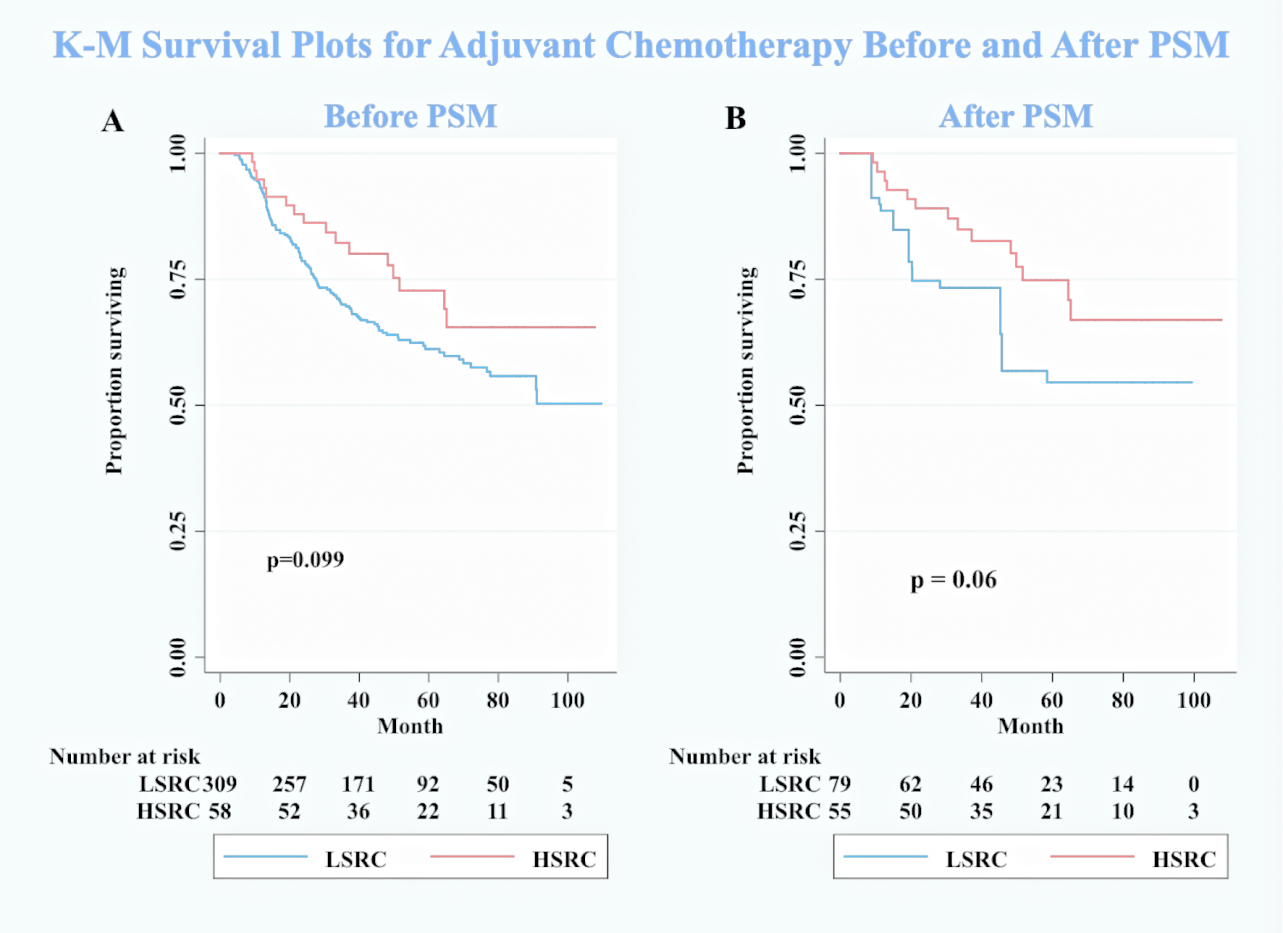
Kaplan–Meier survival curves for patients with adjuvant chemotherapy between low-proportion signet ring cells LSRC and high-proportion signet ringcells. **(A)** Before PSM. **(B)** After PSM.

In subgroup analyses (**Table 2**), although the crude model showed a significant association in the female subgroup (cHR = 0.35, 95%CI = 0.17, 0.73), the effect measure turned null in the multivariable analysis (aHR = 0.50, 95%CI = 0.19, 1.33); a significant interaction was observed between gender and the proportions of signet ring cells (*p*-interaction <0.01 for gender). The results showed that the impact of the proportions of signet ring cells was more substantial among individuals at the early stage (aHR = 0.10, 95%CI = 0.03, 0.32, *p*-interaction <0.01 for stage). Moreover, patients with HSRC at the early stage in **Figure 2** and N0 stage in **Figure 3** continued to demonstrate significantly increased overall survival rates compared to patients with LSRC. In **Figure 4**, HSRC had an insignificant advantage of survival than LSRC in the adjuvant chemotherapy group. We assessed the results of matching by imbalance testing and producing corresponding figures. The mean bias decreased from 29.9 to 4.9, with a *p*-value from 0 to 0.569. The standardized bias across covariates was close to zero after matching. A mean bias of <5% after matching was considered to indicate a good balance (**Supplementary Table S1**). The curves of Kdensity Pscore and propensity Pscore showed a high-fitting degree after matching.

## Discussion

To our knowledge, this is the first study to examine the effect of the proportion of signet ring cells on prognosis in GC in a Chinese population. Overall, our study suggests that HSRC in GC was associated with better survival, and the results showed an interaction between the proportion of signet ring cells and gender and stage at diagnosis in relation to overall survival.

A recent multicenter study investigated similar research questions as ours. The survey of Bencivenga et al. (*N* = 173) including people from three European centers reported that the percentage of signet ring cells was inversely related to tumor aggressiveness (10–90 *vs*. ≥90%: HR = 2.08, 95%CI = 1.01, 4.29; ≤10 *vs*. ≥90%: HR = 2.38, 95%CI=1.05, 5.41). However, their study population and designs are different from ours, suggesting that their outcomes may be less generalizable to Chinese GC patients due to methodological heterogeneities, for example, they did not exclude the effect of R1 and R2 resection on survival, and the analysis included participants with incomplete lymph nodes removed. In our study, we excluded the factor cited above, which could significantly affect survival, and had an adequate sample size, which led to accurate analysis and powerful evidence for the effects of signet ring cells in GSRC. Furthermore, we also examined the survival of gastric cancer patients with four classifications of signet ring cell amount and found the appropriate grouping. On the other hand, our findings were not in line with conclusions from some previous studies, for example, Nafteux et al. enrolled 114 GSRC patients (HSRC = 32 and LSRC = 82) in Belgium and found that HSRC had a lower 5-year cancer-specific survival (16 *vs*. 36%, *P* = 0.1) and lower median survival (19.1 *vs*. 28.7 months) than LSRC (22). One potential reason may be that the gastroesophageal junction cancer has specifically pathological characteristics compared to gastric cancer, such as squamous cells in esophageal cancer, and the sample size was too small in that study to conduct a precise analysis.

Interestingly, we noticed the robust results of better survival in the HSRC group after balancing the key factors by PSM. We have some speculations about the mechanisms behind the association patterns. The first possible explanation might be that the LSRC group (<50% signet ring cell and >50% adenocarcinoma) mixed up the adverse features of lymph node metastases in intestinal type, peritoneal seeding in diffuse type, and chemoradiotherapy resistance in signet ring cell type, which led to a poor prognosis (23). Another is that signet ring cells at the early stage are associated with a less aggressive feature. When the signet ring cell has invaded the submucosa, even serosal, layer, it will promote tumor invasion, lymph node metastasis, and peritoneal seeding, increase the chemoresistance, and worsen the prognosis (24).

One subgroup analysis finding suggests that patients with N0 stage can get survival benefits from HSRC in GC. A high portion of T1 stage (77.6%) in HSRC may be the reason that leads to this result (10). The mechanism behind this result is uncertain. Future studies on the current topic are therefore recommended.

Another unanticipated finding was that the HSRC patients seemed to get benefits from adjuvant chemotherapy in the survival analysis. However, this finding did not achieve statistical significance in our study. It has been commonly observed with that GSRC is less sensitive to chemotherapy, by comparison to intestinal type of GC (25). A comparison of the findings with those of other studies included 899 GSRC and confirmed that preoperative (HR = 1.062, 95%CI: 0.819–1.376) and postoperative (HR = 0.873, 95%CI: 0.708–1.077) chemotherapy did not significantly impact on the survival of GSRC (24). The shortage of study on the above-mentioned topic is due to the fact that the inclusion criteria were too wide. It included any diffuse-type gastric cancer with identified signet ring cells (percentage not specified) as a GSRC. This criterion may lead to inaccurate conclusions. However, Heger et al. observed that neoadjuvant chemotherapy was an independent prognostic factor (HR = 0.66, *p* = 0.023) and improved the survival (median survival: 28.5 *vs*. 14.9 months, *p* < 0.001) in 310 patients with esophagogastric signet ring cell cancer (26). The role of the signet ring cells in the chemotherapy of GC is still uncertain, and the conclusions are varied. In our study, stratification according to the signet ring cell component is conducted to explore the chemosensitivity and response to adjuvant chemotherapy. Although our statistical results reported that adjuvant chemotherapy may not improve the survival of HSRC patients, the survival curves showed a hopeful trend. Further biological studies, drug discovery, and new treatment strategies are required to improve the prognosis of GSRC in the future.

### Strengths and Limitations

Our study has some merits in design and analysis. With an 8-year follow-up time span, our study highlights the big effect of a high proportion of signet ring cell on the survival of GSRC patients. This study also analyzed the effect of nodal stage and adjuvant chemotherapy on survival in multi-dimensions, with robust statistics such as univariate analysis and multivariable Cox proportional hazard model, that could greatly diminish the impact of confounders and explore potential effects in certain group. Furthermore, we chose a propensity score-matched analysis to reduce or eliminate the effects and potential bias of confounders since the baseline characteristics often differ systematically between the groups. This method allowed us to do a better comparison of characteristics between the LSRC and HSRC groups. Adequate lymphadenectomy was conducted, where 96% patients removed >16 lymph nodes. It avoided the bias by surgical technique factor. We adjusted for several metabolic indicators (obesity, diabetes mellitus, hypertension, and high cholesterol) to diminish the impact of such residual confounding.

Although this study has the aforementioned strengths, it still has several limitations. One limitation was the lack of information about the specific regimen of chemotherapy. Another limitation of this study was that we cannot include disease-free survival as outcome due to the lack of recurrence and metastasis data.

## Conclusion

In conclusion, this research suggests that higher proportions of signet ring cells are associated with better overall survival, particularly if diagnosed at an early stage or N0 stage. When it comes to GSRC with low proportions of signet ring cells, combined modality treatments (*e*.*g*., postoperative chemoradiotherapy or perioperative chemotherapy) should be taken into consideration, for a better prognosis, by clinicians when making medical decisions. Further prospective study is needed to confirm our findings and access optimal methods of tissue diagnosis on GC with signet ring cells and tailored treatment for a long-term prognosis.

## Data Availability Statement

The original contributions presented in the study are included in the article/**Supplementary Material**. Further inquiries can be directed to the corresponding author.

## Ethics Statement

Ethical approval was obtained through the Independent Ethics Committee of the National Cancer Center/Cancer Hospital, Chinese Academy of Medical Science, and Peking Union Medical College, and all participants gave written informed consent.

## Author Contributions

YL contributed to conceptualization, methodology, validation, formal analysis, investigation, writing—original draft, writing—review and editing, and visualization. YZ contributed to conceptualization, investigation, writing—review and editing, and supervision. QX contributed to investigation, writing—review and editing, and supervision. ZZ contributed to methodology, validation, formal analysis, writing—review and editing, and visualization. YT contributed to conceptualization, project administration, investigation, writing—review and editing, and supervision. All authors contributed to the article and approved the submitted version.

## Funding

This work was funded by the Chinese Academy of Medical Science/Peking Union Medical College (grant number 2019-1002-80) and the National Natural Science Foundation of China (grant number 82072734). These funding sources had no role in the design of this study and will not have any role during its execution, analyses, interpretation of the data, or decision to submit results.

## Conflict of Interest

The authors declare that the research was conducted in the absence of any commercial or financial relationships that could be construed as a potential conflict of interest.

## Publisher’s Note

All claims expressed in this article are solely those of the authors and do not necessarily represent those of their affiliated organizations, or those of the publisher, the editors and the reviewers. Any product that may be evaluated in this article, or claim that may be made by its manufacturer, is not guaranteed or endorsed by the publisher.

## Supplementary Material

Supplementary Figure 1 Kaplan–Meier survival curves for differently proportional signet ring cell in gastric cancer. Minority, <10% signet ring cells; partialness, 10–50% signet ring cells; majority, 50–90% signet ring cells; total, >90% signet ring cells.
