# Supplementary figures and images for: Prognostic Significance of Signet Ring Cells in Gastric Cancer: The Higher Proportion, The Better Survival
Source: Front Oncol. 2021 Nov 9;11:713587. doi: 10.3389/fonc.2021.713587 (PMC8630623; doi:10.3389/fonc.2021.713587)

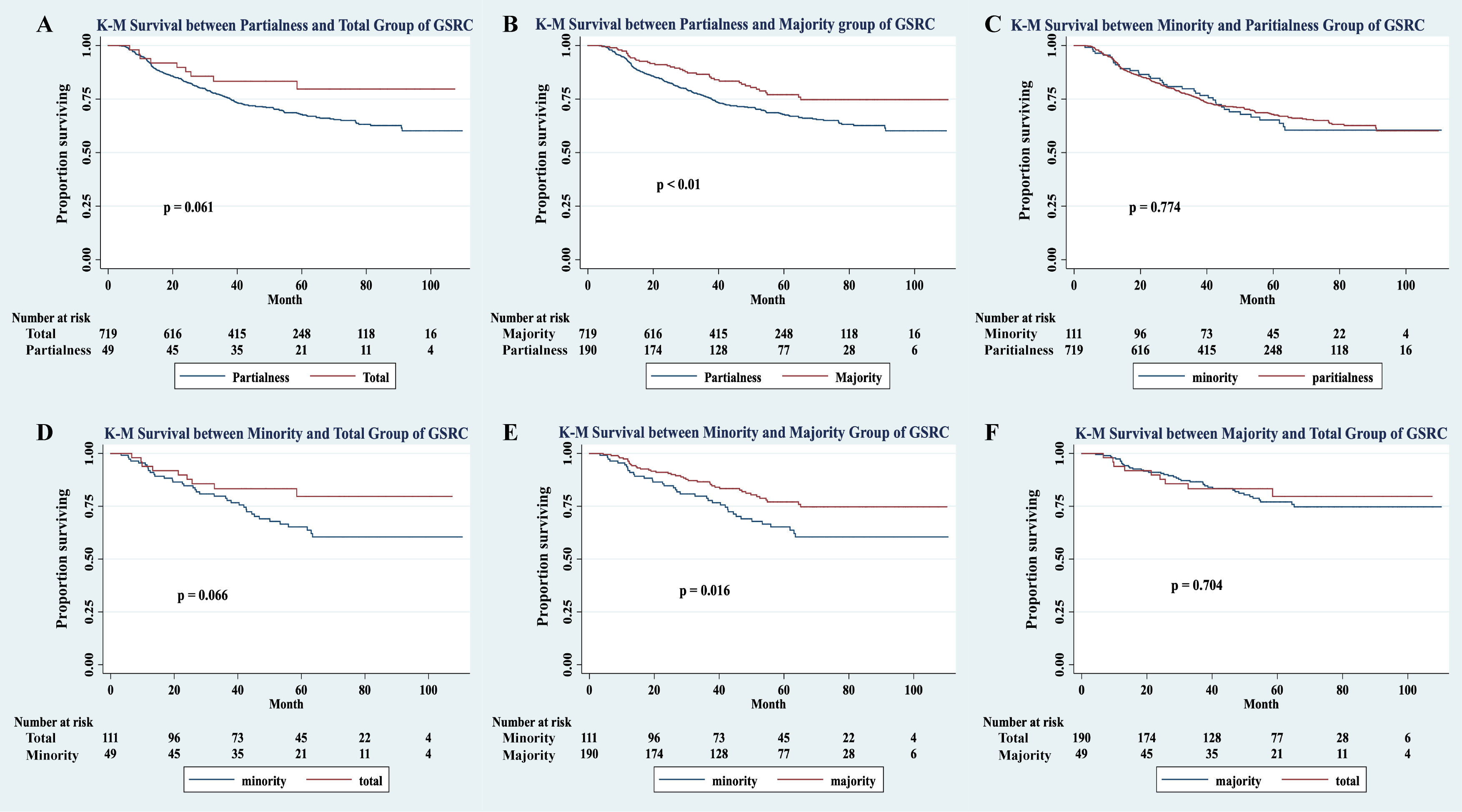

Supplement: Supplementary Figure 1 — Kaplan–Meier survival curves for differently proportional signet ring cell in gastric cancer. Minority, <10% signet ring cells; partialness, 10–50% signet ring cells; majority, 50–90% signet ring cells; total, >90% signet ring cells. [file Image_1.tif]
