# Supplementary material for: Prognostic Significance of Signet Ring Cells in Gastric Cancer: The Higher Proportion, The Better Survival
Source: Front Oncol. 2021 Nov 9;11:713587. doi: 10.3389/fonc.2021.713587 (PMC8630623; doi:10.3389/fonc.2021.713587)
Supplement: Supplementary file 2 [file Table_1.docx]

**Supplementary TABLE S1. PSTEST Assesses Balance in the Matched Variables**

| **Variable** | **Unmatched**  **Matched** | **Mean** | | **%bias** | **%reduct**  **\|bias\|** | **T-test**  **P-value** |
| --- | --- | --- | --- | --- | --- | --- |
|  |  | **Treated** | **Control** |  |  |  |
| **Age** | U | 1.30 | 1.41 | -21.6 | 92.3 | **0.004** |
|  | M | 1.29 | 1.28 | 1.7 |  | 0.844 |
| **Histology Differentiation** | U | 1.37 | 1.05 | 54.0 | 95.9 | **0.000** |
|  | M | 1.31 | 1.30 | 2.2 |  | 0.846 |
| **Nerves Invasion** | U | 1.98 | 1.69 | 35.8 | 82.3 | **0.000** |
|  | M | 1.97 | 1.92 | 6.3 |  | 0.491 |
| **Stage** | U | 20.18 | 23.17 | -32.5 | 80.3 | **0.000** |
|  | M | 19.98 | 20.57 | -6.4 |  | 0.504 |
| **Tumor Site** | U | 2.49 | 2.35 | 16.3 | 93.6 | **0.033** |
|  | M | 2.48 | 2.47 | 1.0 |  | 0.902 |
| **c.Stage#c.Stage** | U | 496.79 | 615.66 | -29.6 | 79.0 | **0.000** |
|  | M | 487.94 | 512.89 | -6.2 |  | 0.511 |
| **c.Stage#c.Age** | U | 27.38 | 33.15 | -30.2 | 93.5 | **0.000** |
|  | M | 26.81 | 27.19 | -1.9 |  | 0.826 |
| **c.Stage#c.Histology Differentiation** | U | 27.16 | 24.06 | 19.2 | 35.5 | **0.001** |
|  | M | 25.42 | 27.42 | -12.4 |  | 0.284 |
|  |  | **Ps R2** | **Mean Bias** | | **MedBias** | **P-value** |
| **Total** | U | 0.100 | 29.9 | | 29.9 | 0.000 |
|  | M | 0.010 | 4.8 | | 4.2 | 0.569 |

Note: Boldface indicates statistical significance (p<0.05).
